# Mental Health and Psychosocial Functioning in Recently Separated U.S. Women Veterans: Trajectories and Bi-Directional Relationships

**DOI:** 10.3390/ijerph18030935

**Published:** 2021-01-22

**Authors:** Karen A. Lawrence, Dawne Vogt, Adam J. Dugan, Shawn Nigam, Emily Slade, Brian N. Smith

**Affiliations:** 1 College of Social Work, University of Kentucky, Lexington, KY 40506, USA; 2 National Center for PTSD Women’s Health Sciences Division, VA Boston Healthcare System, Boston, MA 02130, USA; Dawne.Vogt@va.gov (D.V.); Brian.Smith12@va.gov (B.N.S.); 3 Department of Psychiatry, Boston University School of Medicine, Boston, MA 02118, USA; 4 Department of Biostatistics, College of Public Health, University of Kentucky, Lexington, KY 40536, USA; adam.dugan@uky.edu (A.J.D.); shawn.nigam@uky.edu (S.N.); Emily.Slade@uky.edu (E.S.)

**Keywords:** posttraumatic stress disorder, depressive disorder, sexual harassment, veterans’ health, occupational health, functional impairment

## Abstract

Prior research on the relationship between veterans’ mental health and psychosocial functioning has primarily relied on male samples. Here, we investigated prospective longitudinal relationships between mental health and psychosocial functioning in 554 female Iraq and Afghanistan War veterans who were surveyed three times between two- and seven-years following separation from service. Mixed effects modeling revealed that increasing depression and posttraumatic stress disorder (PTSD) severity predicted declines in work functioning. Increasing PTSD severity predicted declining parental functioning and worsening depression predicted a decline in relationship functioning. In turn, decreased work and intimate relationship functioning predicted increased PTSD and depression symptom severity suggesting bi-directional effects between mental health and psychosocial functioning. An examination of the effect of deployment stressors on psychosocial functioning revealed that deployment sexual harassment was the strongest predictor of decreased psychosocial functioning across all domains. Evidence for the reciprocal nature of relationships between mental health and psychosocial functioning underscore the need for treatment targeted at PTSD and depression, as well as work and relationship functioning to improve outcomes for women veterans.

## 1. Introduction

Prospective research is needed to better understand U.S. women veterans’ mental health and functional challenges. While research shows that the majority of veterans report a high level of post-military psychosocial functioning, some veterans may face challenges with employment as well as relationship and family functioning [1,2,3,4]. For example, a study conducted in 2019 found that among women veterans between 35 and 44 years old, 5.0% of women veterans were unemployed compared to 2.9% of non-veteran women [5]. The greater rate of unemployment for female veterans is of particular concern given that unemployment is a risk factor for homelessness among women veterans [6], who have been found to be at a higher risk of being homeless than their non-veteran counterparts in prior research [7]. In addition to their risk for poor occupational outcomes, prior studies have revealed that post-9/11-era women veterans differ from both male counterparts and nonveteran women with respect to their family functioning [4,8,9]. For example, women veterans are more likely to be divorced and remain divorced relative to veteran men and non-veteran women [8]. Importantly, women are projected to comprise 16.3% of the veteran population by the year 2042 [9,10] and prospective research on recently returned women veterans’ psychosocial functioning is needed to inform providers, programs, and policies, and to benefit the next generation of women veterans.

The majority of studies that have investigated the relationship between veterans’ mental health and post-military psychosocial functioning have been carried out with predominantly male samples [11,12,13,14,15,16,17,18,19,20,21,22] and focused mainly on veterans who served in the post-9/11 wars in Afghanistan and/or Iraq [Operations Enduring and Iraqi Freedom (OEF/OIF)]. Typically, these studies have been cross-sectional with few evaluating longitudinal outcomes. Of the psychosocial functioning domains that have been assessed in this research, relationship and family functioning have been the most addressed, followed by work functioning as reviewed below. Of note, the literature on veterans’ psychosocial functioning is heterogeneous with respect to the measurement instruments used to assess work, relationship, and family functioning outcomes. In the next section we review findings in male-focused studies, followed by the more limited literature on women veterans.

### 1.1. Mental Health and Psychosocial Functioning in Men

Studies that have evaluated work functioning outcomes in male veterans have tended to focus on the effects of PTSD, major depressive disorder (MDD), and alcohol dependence on these outcomes. Cross-sectional studies have shown an association between PTSD diagnosis and multiple aspects of impairment in work functioning [11], even when controlling for other Axis I disorders [17]. In addition, there is some evidence that PTSD is associated with deterioration in work role functioning over time [12]. Diagnoses of MDD and alcohol dependence have also been found to be associated with occupational impairment [11], and one study found that individuals who experience improvements in depression were more likely to remain continuously employed [19]. Finally, a study from the parent project of the current study [23], which reported on gender differences in the first two time points of this longitudinal investigation of veterans’ mental health and functioning, found that greater PTSD symptom severity directly predicted downstream impairment in work functioning and mediated the impact of warfare exposure on work impairment. However, neither depression nor deployment sexual harassment were associated with work functioning, either directly or indirectly.

Studies that have examined associations with relationship and family functioning outcomes in male veteran samples have typically focused on the effects of PTSD diagnosis rather than MDD and alcohol dependence. In cross-sectional studies, PTSD symptoms have been found to be associated with impairments in intimate relationship functioning and satisfaction [14,16,19,23], and one study showed an association between hazardous drinking and relationship distress [21]. Longitudinally, increasing trauma symptoms, but not depression nor alcohol use, have been shown to predict lower intimate relationship functioning [14,23] and PTSD symptom severity has been found to mediate the relationship of both warfare exposure and deployment sexual harassment with relationship impairment [23].

PTSD has also been found to be associated with impairment in parental functioning [13] and lower family cohesion among male veterans [18]. In addition, research indicates that depression [21,22], and alcohol misuse [22] are associated with negative family functioning. Longitudinally, increases in PTSD symptoms have been found to predict greater perceived parenting challenges independent of relationship functioning [14]. In addition, PTSD symptom severity has been shown to directly predict parental impairment [4] and to mediate the relationship between warfare exposure and parental impairment [23]. Furthermore, research indicates that impairment in parental functioning is directly predicted by deployment sexual harassment in men [23].

### 1.2. Mental Health and Psychosocial Functioning in Women

The literature on women’s mental health and psychosocial functioning is much more limited, which is problematic given that the research that is available suggests that women veterans differ from veteran men with respect to both their experience of deployment stressors and their post-military mental health [24,25,26,27]. For example, research indicates that relative to veteran men, women veterans are more likely to experience lifetime PTSD [26] and depressive disorders and less likely to experience substance use disorders [24,26]. Research has also demonstrated that women are more likely to experience military sexual trauma and less likely to experience combat [24,28,29]. For example, recent meta-analytic findings revealed that an estimated 38.4% of women compared to 3.9% of men reported experiencing military sexual trauma [28]. However, only a few studies have examined the relationship between psychosocial functioning and mental health specifically among women veterans, and fewer still have accounted for the effects of deployment stressors.

Within the developing literature on women veterans’ post-military psychosocial functioning, PTSD symptom severity has been associated with poorer quality of life in both work [29] and family [30] domains of psychosocial functioning. Prior research from the parent project [4,23] of the current study found that PTSD was not associated with work functioning in women, yet depression was, and depression mediated the relationship between several deployment stressors and greater impairment in work functioning. In addition, alcohol misuse severity was found to mediate the association between deployment sexual harassment and post-military impairment in intimate relationship functioning. Interestingly, both depression and PTSD were associated with intimate relationship functioning and both were found to mediate the association between exposure to deployment stressors and greater post-military intimate relationship impairment. Finally, PTSD was the only mental health condition that predicted parental functioning, and PTSD symptom severity was found to mediate the association between several deployment stressors and greater impairment in parental functioning. Given the limited research on women veterans’ mental health and psychosocial functioning, our goal for the present study was to investigate the following research questions in a sample of U.S. women veterans who experienced a deployment in support of OEF/OEF during their military service:

1. How does women veterans’ mental health and psychosocial functioning change over time?
2. How do deployment stressors and changes in mental health symptom severity affect changes in women veterans’ psychosocial functioning over time?
3. Is there a bi-directional relationship between women veterans’ mental health symptom severity and psychosocial functioning?

Although the current investigation drew from a dataset that has been used in prior research [4,23], the current study benefited from the availability of an additional data collection, which allowed for the examination of trajectories over a seven year period, as well as the novel investigation of how longitudinal changes in mental health (T1 to T2) predict downstream longitudinal changes in psychosocial functioning (T2 to T3) and the exploration of bi-directional relationships between mental health symptom severity and psychosocial functioning.

## 2. Materials and Methods

Data for this study were drawn from the Veterans’ Work and Family Functioning study, which was designed to examine veterans’ post-military readjustment and quality of life with regard to work and family domains. Methods of ascertainment, recruitment, and assessment have been described in detail elsewhere [4,23]. A brief overview is provided here.

### 2.1. Participants and Procedure

**Figure 1:**
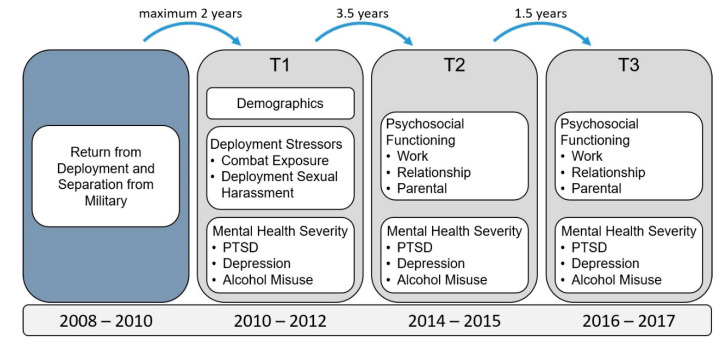
Study timeline.

OEF/OIF Veterans within two years of military service completion (2008–2010) were identified using a Department of Defense (DoD) roster. Random sampling was used, and the sample was stratified by deployment component (50% deployed from Active Duty, 50% from National Guard/Reservist units) and gender, with women oversampled at 50%. As shown in Figure 1, veterans completed surveys at three time points over seven years: (1) T1 was completed by 1046 veterans within two years of separation from the military, (2) T2 was completed three and a half years later by 522 veterans, and (3) 1.5 years subsequent to T2, 455 veterans completed the T3 survey. The study sample here is comprised of the women veteran subsample (N = 554 at T1). This study was approved by the Institutional Review Board at VA Boston Healthcare System.

Cohort retention analyses showed that, of the 554 women veterans at T1, 272 responded at T2 (49%), and 230 at T3 (41.5%). In order to detect systematic longitudinal differences between responder and non-responder groups, we tested for demographic and clinical differences between responders and non-responders using Pearson’s χ2 tests for categorical variables and Pearson’s correlation coefficient for continuous variables. We compared those who responded at T1 and T2 to those who responded at T1 but not T2 and found that attrition was associated with being younger (*p* < 0.0001), Non-White (*p* = 0.042), having a lower level of education (*p* = 0.003), and any mental health diagnosis (*p* < 0.0001). There were no significant demographic or clinical differences between responders and non-responders at T3.

### 2.2. Measures

#### 2.2.1. Mental Health Conditions

PTSD symptom severity was measured at T1, T2, and T3 using the PTSD Checklist–Military Version (PCL-M) comprised of 17 items with a 5-point response scale ranging from 1 (not at all) to 5 (extremely) to indicate the degree to which a particular symptom was bothersome over the prior three months. The score range is 17–85 with higher scores indicating worse severity. Studies have shown that the PCL-M as well as the civilian version have strong psychometric characteristics [31,32,33,34]. Among veteran samples, the PTSD Checklist showed good internal consistency with alphas above 0.80 and 0.75 [34], 0.97, and 0.96 [33] as well as 0.96 [32]. In the current study, we also found good internal consistency (current study α = T1: 0.96, T2: 0.96, T3: 0.96). Additionally, good convergent validity was shown by several studies utilizing a veteran sample [32,33,34]. Sensitivity and specificity scores ranged between 0.81 [35] and 0.83 [33]. In addition to using severity scores as continuous variables, some analyses used cut-off scores in which probable PTSD was operationalized as a score of 35 or greater on the PCL-M and meeting the DSM-IV requirement of one re-experiencing item, three numbing and avoidance items, and two hyperarousal items being at least moderately bothersome.

Depression symptom severity was measured at T1, T2, and T3 using the 7-item Beck Depression Inventory-Primary Care (BDI-PC). The 5-point response scale ranges from 1 (strongly disagree) to 5 (strongly agree), indicating how much the participant experienced a particular symptom over the prior 3 months. The scale range is 7–35 with higher scores indicating worse severity. Within different medical patient samples, the reliability scores for the BDI-PC scale are high, with coefficient alphas ranging between 0.86 [36,37] and 0.88 [38] (current study α = T1: 0.89, T2: 0.91, T3: 0.91). In addition to using severity scores as continuous variables, some analyses used cut-off scores in which probable depression was operationalized as a score of 4 or greater on the BDI-PC following the finding that this score range yielded the maximum clinical efficiency with 82% sensitivity and specificity [37].

Alcohol misuse was measured using the Alcohol Use Disorders Identification Test for Consumption (AUDIT-C). Scores can range from 0 to 12 with higher scores indicating worse severity and lower scores indicating little/no symptoms. Among various populations, such as patients, the AUDIT has demonstrated good internal reliability (0.75–0.97) [39,40]; in the current study, we also found good internal consistency (α = T1: 0.81, T2: 0.78, T3: 0.76). The AUDIT-C has good validity [40,41], high sensitivity and specificity [41,42], and is a valid tool for assessing alcohol misuse among military populations [43] including women veterans [44]. In addition to using severity scores as continuous variables, some analyses used cut-off scores in which probable alcohol use disorder was operationalized as a score of 2 or greater on the CAGE, a 4-item screening test used to identify problems with alcohol use [45,46]. The CAGE has been shown to have high test-retest reliability [47], good concurrent validity and psychometric properties using a cut-off score of 2 or greater [48,49]. Finally, probable traumatic brain injury (TBI) was assessed using an adapted version of the Brief Traumatic Brain Injury Screen [50].

#### 2.2.2. Psychosocial Functioning

The Inventory of Psychosocial Functioning (IPF) was administered at T2 and T3 to assess work, intimate relationship, and parental functioning. Overall, the IPF has shown to be a valid and reliable measurement tool [51,52,53] with an alpha coefficient of 0.93 for the overall scale [54]. Subscales have also demonstrated robust internal consistency with alphas ranging from 0.79 to 0.90 [52,53] and from 0.80 to 0.90 [54]. All subscales use a 7-point Likert response format on which 1 = never and 7 = always. In the present study, items were coded prior to calculating the summed score so that higher sum scores indicate better functioning and less impairment.

Work functioning was assessed using a 21-item subscale from the IPF [54] with items such as: *I had trouble showing up on time for work* and *I took responsibility for my work*. Summed scores could range from 21 to 147 with higher scores denoting better functioning. The work functioning scale has shown good reliability with an alpha of 0.92 [51] (current study α = T2: 0.90, T3: 0.89). Intimate relationship functioning was assessed using an 11-item subscale from the IPF with times such as: *I showed interest in my spouse or partner’s activities* and *I was patient with my spouse or partner*. Summed scores could range from 11 to 77 with higher scores meaning better functioning. This scale has been shown to have good reliability of 0.86 [51] (current study α = T2: 0.91, T3: 0.91). Parental functioning was assessed using a 10-item subscale from the IPF with items such as: *I had trouble communicating with my children* and *I was a good role model for my children*. Summed scores could range from 10 to 70 with higher scores meaning better functioning. This scale has been shown to be reliable with an alpha of 0.85 [51] (current study α = T2: 0.94, T3: 0.90).

#### 2.2.3. Deployment Stressors

The Deployment Risk and Resilience Inventory-2 (DRRI-2) has been demonstrated to be reliable and valid in veteran samples [55,56]. Scales were administered at T1 and higher scores indicated greater stress exposure. Combat exposure was measured with 17 items on a 6-point response scale wherein 1 = never and 6 = daily or almost daily, and items specified deployment activities such as: *I went on combat patrols or missions* and *I personally witnessed someone from my unit, or an ally unit being seriously wounded or killed*. The deployment sexual harassment measure included 8 items on a 4-point response scale ranging from 0 = never to 3 = many times, with items such as: *The people I worked with made crude and offensive sexual remarks directed at me, either publicly or privately* and *The people I worked with physically forced me to have sex*. While the term “deployment sexual harassment” is used in the current study, note that the measure used includes items ranging from harassment to assault and is therefore representative of both harassment and assault components of military sexual trauma.

### 2.3. Analyses

Descriptive analyses were performed at baseline (T1). Less than 10% of observations were missing and pairwise exclusion was used for descriptive analyses which were performed using SPSS version 26 [57].

For longitudinal modeling, summed scale scores were used, and statistical significance was set at *p* ≤ 0.05. Linear mixed effect regression models with maximum likelihood (ML) estimation were used to model trajectories of mental health and psychosocial factors. Random intercepts were included for each individual to account for the correlation that exists between repeated observations made on the same individual. ML estimation was used since it is robust to missingness and allows for all available observations at each time point to be included. The residuals of all mixed effect models were examined for departures from normality; in the case of a significant departure from normality, an appropriate transformation was applied to the outcome.

In order to investigate the effects of PTSD, depression, and alcohol misuse at T1 and T2 on work, relationship, and parental functioning at T2 and T3, change scores from T1 to T2 were calculated for each mental health predictor and these predictors were included in separate adjusted and unadjusted multivariable regression models. Subsequently, all predictors were combined into a single model (one unadjusted model and one adjusted for combat exposure and deployment sexual harassment). Bi-directional effects were examined from T2 to T3 using separate linear regression models for each mental health and psychosocial variable combination. Change scores from T2 to T3 were calculated for each psychosocial predictor and these predictors were included in separate regression models, each adjusted for the severity level of the mental health variable at T2. The modeling was performed with and without adjustment for deployment stressors. All change scores were calculated with later time-point subtracted from earlier time-point. All linear modeling analyses were performed using R programming language, version 3.6.3 [58]. All mixed models were fit using the R package lme4, version (1.1–21).

## 3. Results

Women veterans in the current study reported an average age of 34.1 years (SD = 10.0). The majority reported their race as White (71.5%) or Black (18.2%) and 13.6% reported being of Hispanic ethnicity. Approximately half of the sample reported having a post-high school education (51%) with nearly 43% having completed a 4-year college degree or graduate level of education. Over half reported being married or partnered (53.4%) and nearly half reported having children (46.6%). The majority of women reported having served in the army (65.2%) with over half describing their military job role as combat arms/support (54.6%) while others designated their role as service support.

Regarding deployment stressors, the majority of women (70.3%) reported at least one instance of exposure to combat. The most frequently endorsed combat exposure items were: *I was exposed to hostile incoming fire* (52.5%) and *I went on combat patrols or missions* (41.9%). Just under half of women (45.1%) reported at least one instance of sexual harassment/assault during deployment. The most commonly endorsed items included: *The people I worked with made crude and offensive sexual remarks directed at me, either publicly or privately* (34.6%), *The people I worked with spread negative rumors about my sexual activities* (29.4%), and *The people I worked with tried to talk me into participating in sexual acts when I didn’t want to* (16.9%).

The following prospective results are based upon deployment stressor outcomes assessed at T1. Mental health assessments were conducted at T1, T2, and T3 and psychosocial functioning was measured at T2 and T3 (Figure 1). At baseline (T1), 54.2% (292/539) of women veterans screened positive for probable depression, 25.6% (139/543) for probable PTSD, and 12.1% (66/547) for probable alcohol use disorder. Regarding deployment stressors, 45.1% (242/537) reported experiencing some level of sexual harassment and 70.3% (371/528) reported some level of exposure to combat.

### 3.1. Trajectories of Mental Health and Psychosocial Functioning

Mean depression symptom severity decreased (improved) between T1 and T2 (B = −1.776; 95% CI: −2.503, −1.054; *p* < 0.001) and the decrease was maintained from T2 to T3 (B = −1.447; 95% CI: −2.222, −0.675; *p* < 0.001). Despite this decrease, depression scores remained in the moderate clinical range (T1 Mdn = 22.5, IQR = 16–27; T2 Mdn = 20, IQR = 12–25; T3 Mdn = 20, IQR = 13–26). The proportion of women screening positive for a probable depression diagnosis also decreased significantly over time with positivity rates of 54.2% at T1, 39.4% at T2, and 45.1% at T3, such that the odds of screening positive for probable depression decreased at T2 relative to T1 (OR = 0.897, 95% CI: 0.842, 0.942; *p* < 0.001) and at T3 relative to T1 (OR = 0.936; 95% CI: 0.881, 0.994; *p* = 0.032). Work functioning became increasingly impaired for the sample as a whole, as indicated by a decrease in mean level of functioning from T2 to T3 (B = −2.289; 95% CI: −4.217, −0.352; *p* = 0.021). PTSD, alcohol misuse, intimate relationship functioning, and parental functioning did not change significantly over time for the sample as a whole.

### 3.2. Effects of Mental Health and Deployment Stressors on Psychosocial Functioning

We examined the individual effects of combat exposure and deployment sexual harassment measured at T1, as well as T1 to T2 changes in PTSD, depression, and alcohol misuse severity, on changes in psychosocial functioning from T2 to T3 in unadjusted models as well as models adjusted for combat exposure and sexual harassment during deployment (Table 1). In unadjusted models, all three psychosocial domains decreased significantly due to combat exposure and deployment sexual harassment with sexual harassment having the strongest effect, by far. Work functioning from T2 to T3 decreased significantly due to T1 to T2 increases in PTSD and depression. Decreased T2 to T3 relationship functioning was associated with increased T1 to T2 depressive symptoms and a T2 to T3 decrease in parental functioning was associated with a T1 to T2 increase in PTSD symptoms. These relationships persisted even after adjusting for combat exposure and deployment sexual harassment. In addition, after adjustment for deployment stressors, a relationship emerged wherein increases in T1 to T2 PTSD symptom severity was associated with decreases in relationship functioning between T2 and T3.

Next, we examined the effects of T1 to T2 changes in the three mental health predictors, simultaneously, on T2 to T3 changes in functioning within a given psychosocial domain, in unadjusted and adjusted models (Table 2). In unadjusted models, decreases in both work and relationship functioning were associated with increases in depression symptoms. In adjusted models, the associations between work and relationship functioning and depression persisted while additional relationships emerged in models of work and parental functioning. Decreases in work functioning were associated with increases in PTSD symptoms in addition to depression symptoms. Parental functioning was negatively impacted by a T1 to T2 increase in PTSD symptoms. Overall, increases in depression and PTSD were the strongest predictors of declines in psychosocial functional impairment. Greater combat exposure predicted decreased work and relationship functioning but not parental functioning. Higher levels of deployment sexual harassment predicted decreases in functioning across all three psychosocial domains and had the strongest association with decreased psychosocial functioning of all predictors tested.

### 3.3. Effects of Psychosocial Functioning on Mental Health Symptom Severity

Having established the impact of changes in mental health symptom severity on downstream psychosocial functioning, we set out to investigate the potential for bi-directional effects between mental health symptom severity and psychosocial functioning by examining the effects of psychosocial functioning on downstream mental health symptom severity. Linear regression modeling was performed for each mental health and psychosocial variable combination. The effects of T2 to T3 changes in psychosocial functioning predictors on T3 mental health severity was examined after adjusting for mental health severity at T2. The modeling was performed with and without adjustment for deployment stressors. Decreased T2 to T3 work functioning was associated with higher T3 PTSD symptom severity (B = 0.390; 95% CI: 0.239, 0.541; *p* < 0.001) and T3 depression symptom severity (B = 0.186; 95% CI: 0.103, 0.268; *p* < 0.001). Likewise, decreased T2 to T3 relationship functioning was associated with higher T3 PTSD symptom severity (B = 0.361; 95% CI: 0.191, 0.530; *p* < 0.001) and T3 depression symptom severity (B = 0.205; 95% CI: 0.115, 0.295; *p* < 0.001). In other words, as psychosocial functioning scores decreased from T2 to T3 (became worse), resulting in higher change scores of positive values (wherein T3 values were subtracted from T2 values), T3 mental health symptom severity scores increased. These relationships were significant in models with and without adjustment for deployment stressors; the adjusted model values are shown.

## 4. Discussion

The goal of this study was to provide a clearer understanding of the relationship between U.S. women veterans’ post-military psychosocial functioning and mental health. To achieve that goal, we examined the relationship among these factors within a longitudinal study that spanned seven years. To our knowledge, no other study has examined the relationship between post-military trajectories of mental health and psychosocial factors over such an extended period of time. First, we investigated how women veterans’ mental health and psychosocial functioning changed over time. Although longitudinal changes were observed, wherein depression symptom severity decreased over time, depression remained in the moderate severity range and work functioning became increasingly impaired during the study period. The perdurance of moderate depression is concerning as women OEF/OIF veterans are more likely to receive a depression diagnosis than men [27]. Further, in comparison to non-veterans, depression in female veterans has been associated with a number of long-term comorbidities [59] including coronary artery disease [60] as well as respiratory disease, accidents, Alzheimer’s disease, and suicide, which all increase the hazard of mortality [61]. Therefore, additional attention on depression symptom reduction is warranted. The observed decrease in work functioning is also concerning given higher unemployment rates in women veterans compared to non-veteran counterparts [5] especially given that unemployment is a risk factor for homelessness among women veterans [6], and that women veterans have a higher risk of being homeless than non-veteran women [7].

For our second research question, we explored how changes in mental health symptom severity affect changes in psychosocial functioning while accounting for and investigating the effect of deployment stressors. Overall, these findings demonstrate the impact that both depression and PTSD have on women veterans’ psychosocial functioning, with both affecting multiple domains and persisting in severity and highlight the need for increased implementation of early interventions to prevent declines in female veterans’ post-military functioning across work, intimate relationship, and parental domains. The importance of targeting PTSD and depression early is further underscored by the finding that PTSD symptom severity did not change significantly over time and change in depression scores was modest, which suggests that once in place these symptoms are unlikely to improve without treatment.

The finding that decreased work functioning from T2 to T3 was predicted by greater exposure to deployment stressors, with deployment sexual harassment being a particularly strong predictor, underscores the substantial impact that military sexual harassment has on women veterans’ lives and indicates a need for continued efforts focused on screening and treatment focused on deployment sexual harassment.

Worsening depression and PTSD symptoms from T1 to T2 also predicted decreased work functioning, with changes in depression having the stronger association. This finding is consistent with research in civilian samples, which has demonstrated that successful treatment of depression results in improved work performance [62,63,64]. Interestingly, in a prior study, T1 PTSD symptom severity negatively impacted T2 work functioning in men but not women [23]. Our current findings suggest that this association also occurs in women, however, the relationship emerges at a later time-point among women. Although the relationship between deployment stressors, depression, and work functioning in women was identified and emphasized in a prior study [23], current findings suggest that the effect of deployment stressors and depression on work functioning persists over a longer timeframe. These findings are consistent with another study that found that women veterans who endorsed sexual assault during adulthood experienced worse occupational functioning one year later [65]. The finding that the strongest predictors of the decrement in work functioning, deployment sexual harassment and depression, are more prevalent in veteran women than men, support the value of additional support with regard to women’s work functioning.

Although T1 alcohol misuse severity was found to directly predict decreased T2 work functioning in prior research [23], within the context of the current study, which includes an additional time-point, change in T1 to T2 alcohol misuse severity did not significantly predict changes in T2 to T3 work functioning, suggesting that the risk of alcohol use affecting occupational functioning may be of greatest concern at a time more proximal to separation from the military with less potential risk as time since separation grows. This finding supports the importance of early interventions to address risky drinking among female veterans.

Increases in mental health symptom severity and exposure to deployment stressors were also found to be significantly associated with declines in relationship and parental functioning. Previous findings indicated that both T1 depression and PTSD directly predicted T2 relationship functioning; further, both depression and PTSD played a role in mediating the association between T2 relationship functioning and at least one deployment stressor [23]. Our current findings suggest that these associations continue for a longer term. Regarding parental functioning, previous results showed that T1 PTSD was a direct predictor of decreased T2 parental functioning and that T1 PTSD also mediated the relationship between deployment stressors and T2 parental functioning [23]. Our current finding that decreased T2 to T3 parental functioning was predicted by increased T1 to T2 PTSD symptom severity suggests that this relationship also persists longer term. In addition, combat exposure and deployment sexual harassment both predicted declines in relationship functioning and the latter predicted declines in parental functioning as well, suggesting the added value of attending to the role that deployment stressors play in women veterans’ psychosocial functioning.

The finding that nearly half of women in the current study reported at least one instance of deployment sexual harassment or assault, which was within the range of prevalence rates reported elsewhere [24,28], combined with the negative impact that MST had on women’s psychosocial functioning across all domains, also draws attention to the importance of efforts to prevent and treat MST. Literature on civilian and military survivors of sexual harassment and assault has revealed that, following such an experience, many women experience shame, self-blame, and self-doubt, as well as interpersonal issues within relationships including self-trust issues and difficulty trusting others [66,67], which can erode their functioning within multiple life domains if left unaddressed.

Potential bi-directional relationships between mental health symptom severity and psychosocial functioning were investigated in our third research question. To our knowledge, this is the first study to examine reciprocal relationships between recently separated veterans’ psychosocial functioning and mental health severity. The finding that decreased work and intimate relationship functioning predicted higher PTSD and depression symptom severity suggests that occupational and relationship support may be important for PTSD and depression symptom management and recovery in women veterans. Given that the magnitude of effect was strongest for work and relationship functioning predicting PTSD symptom severity, support of health in these domains may have particular potential to benefit PTSD symptom management. Taken together, these findings suggest that a holistic approach that supports social functioning outside of the typical clinical setting has the potential to be mutually beneficial, working synergistically with mental health treatment to support symptom management. Indeed, an employment support program focused on supporting veterans experiencing chronic PTSD [68] has shown promise for improving both PTSD-related functioning and work-related psychosocial functioning [69]. Further research on whether such programs positively impact functioning in other psychosocial domains or other mental health symptoms (e.g., depression symptoms) would be an important addition to the literature.

Alternatively, efforts to integrate job support strategies into workplaces and behavioral health services could potentially improve mental health outcomes in women veterans. Indeed, studies in civilians who have sought treatment for anxiety or depression, suggest that modifications to psychosocial work conditions—such as lower work hours, on-the-job support from colleagues, and increased job control—may have the potential to improve work functioning and prevent job loss [70,71]. Such strategies have been proposed as important policy aims, and not exclusively for workers with a history of psychopathology, but also as a means of keeping workers in the labor force despite health challenges, in general [70]. One potential immediate strategy may be for mental health providers to discuss work setting with clients in order to identify ways to increase resilience in the context of work [71].

Given the finding that decreased intimate relationship functioning predicted worse PTSD and depression symptom severity, with effects being strongest for PTSD, a treatment component focused on intimate relationship functioning should be considered for women veterans engaged in mental health treatment. While some psychotherapeutic interventions focus on interpersonal relationship dynamics, such as Interpersonal Therapy (IPT), others are more specifically trauma-focused, especially those targeting PTSD. Therefore, an assessment of need for psychotherapeutic support around intimate relationship functioning may be warranted for women veterans receiving psychotherapeutic interventions that do not include an interpersonal/relationship component.

Our previous study elucidated the significant mediational effects linking deployment stressors with psychosocial functioning outcomes at the first follow-up via mental health symptom severity [23]. Deployment stressors examined in the current study, combat exposure and deployment sexual harassment, had statistically significant negative associations with T2 to T3 functioning in all three psychosocial domains examined in every model tested. Taken together, these findings suggest that the importance of considering the potential impact of deployment stressors on psychosocial functioning cannot be overstated, and that cascading effects involving mental health and functional impairment should be addressed within both research and clinical therapeutic settings.

The study findings should be considered within the context of several limitations. A common concern with longitudinal study designs is loss to follow up resulting in selection bias. We computed retention rates and analyzed demographic and clinical attributes of women veterans who did and did not complete T2 and T3 surveys. According to our retention analyses, attrition was associated with younger age, non-white race, lower level of education, and having a mental health diagnosis. Therefore, it is possible that participants who continued were more resilient than those who did not continue. Given that, our findings might be selectively skewed to a healthier population due to underrepresentation of a greater severity in mental health symptomatology over time. At the same time, we did detect significant effects of mental health on psychosocial outcomes. It is possible that the detected effects, while strong in several of the models tested, are underestimated. Another limitation is inability to definitively operationalize level of functioning using the IPF subscales due to a lack of published norms for these scales. That being said, all measures had good internal consistency throughout all study time-points. Finally, while prospective data were used in this study, an exception to that was the measurement of deployment stressors; retrospective self-report at T1 was used to assess combat exposure and sexual harassment during deployment. More research on prospective relationships between mental health and psychosocial functioning is needed in representative samples of women veterans.

## 5. Conclusions

Our findings suggest that early screening and treatment for PTSD and depression as well as deployment sexual harassment are critical to women veterans’ current and future mental health and psychosocial functioning, as mental health and psychosocial functioning are inextricably interrelated. Support in psychosocial functioning domains that fall outside of the traditional clinical setting has the potential to reciprocally support mental health functioning such that a feedforward loop between mental health and psychosocial functioning may be created to promote ongoing symptom management and healthy functioning.

## Figures and Tables

**Table 1:** Effects of deployment stressors and T1 to T2 changes in mental health symptom severity on T2 to T3 psychosocial functioning.

**Mental Health Variable**	**Work**		**Relationship**		**Parental**	
	**B (95% CI)**	***p***	**B (95% CI)**	***p***	**B (95% CI)**	***p***
Unadjusted						
PTSD	0.244 (0.067, 0.420)	0.007 *	0.128 (−0.027, 0.283)	0.105	0.178 (0.032, 0.323)	0.017 *
Depression	0.384 (0.126, 0.641)	0.004 *	0.433 (0.188, 0.679)	0.001 *	0.230 (−0.012, 0.473)	0.063
Alcohol Misuse	0.229 (−0.572, 1.029)	0.574	0.135 (−0.597, 0.865)	0.717	0.169 (−0.535, 0.872)	0.636
Combat Exposure	−0.351 (−0.645, −0.058)	0.019 *	−0.377 (−0.596, −0.158)	0.000 *	−0.258 (−0.450, −0.065)	0.009 *
Sexual Harassment	−0.882 (−1.340, −0.424)	<0.001 *	−0.639 (−1.042, −0.235)	0.002 *	−0.459 (−0.787, −0.131)	0.006 *
Adjusted for Covariates						
PTSD	0.338 (0.154, 0.520)	<0.001 *	0.235 (0.068, 0.402)	0.006 *	0.286 (0.135, 0.438)	<0.001 *
Depression	0.397 (0.141, 0.652)	0.003 *	0.471 (0.227, 0.716)	<0.001 *	0.215 (−0.020, 0.451)	0.073
Alcohol Misuse	0.572 (−0.282, 1.425)	0.188	0.449 (−0.316, 1.212)	0.249	0.664 (−0.081, 1.407)	0.080

Note. * *p* < 0.05; *B* = unstandardized regression coefficient.

**Table 2:** Effects of T1 to T2 changes in mental health symptom severity on T2 to T3 psychosocial functioning.

**Mental Health Variable**	**Work**		**Relationship**		**Parental**	
	**B**	**95% CI**	**B**	**95% CI**	**B**	**95% CI**
Unadjusted						
PTSD	0.145	−0.048, 0.337	0.024	−0.152, 0.199	0.156	−0.006, 0.319
Depression	0.320	0.037, 0.603 *	0.386	0.108, 0.666 *	0.157	−0.112, 0.427
Alcohol Misuse	0.171	−0.629, 0.973	−0.100	−0.846, 0.643	−0.031	−0.786, 0.719
Adjusted for Covariates						
PTSD	0.218	0.016, 0.420 *	0.143	−0.047, 0.334	0.280	0.111, 0.450 *
Depression	0.296	0.023, 0.568 *	0.336	0.062, 0.610 *	0.055	−0.200, 0.311
Alcohol Misuse	0.470	−0.387, 1.326	0.190	−0.587, 0.965	0.429	−0.373, 1.229
Combat Exposure	−0.299	−0.591, −0.009 *	−0.343	−0.587, −0.099 *	−0.205	−0.410, −0.001
Sexual Harassment	−0.850	−1.433, −0.267 *	−0.768	−1.317, −0.217 *	−0.762	−1.199, −0.324 *

Note. * *p* < 0.05; *B* = unstandardized regression coefficient.

## Data Availability

These data, which are owned by the Department of Veterans Affairs (VA), are not publicly available. Data have been shared via a Freedom of Information Act Request in the past.
